# Dual action of Dooku1 on PIEZO1 channel in human red blood cells

**DOI:** 10.3389/fphys.2023.1222983

**Published:** 2023-07-10

**Authors:** Aline Hatem, Gwendal Poussereau, Martin Gachenot, Laurent Pérès, Guillaume Bouyer, Stéphane Egée

**Affiliations:** ^1^ Sorbonne Université, CNRS, UMR8227 LBI2M, Station Biologique de Roscoff, Roscoff, France; ^2^ Laboratory of Excellence GR-Ex, Paris, France; ^3^ Sorbonne Université, CNRS, FR2424, Station Biologique de Roscoff, Roscoff, France

**Keywords:** Dooku1, Piezo1, Yoda1, [Ca^2+^]_i_, non-selective cation channels, erythrocyte

## Abstract

PIEZO1 is a mechanosensitive non-selective cation channel, present in many cell types including Red Blood Cells (RBCs). Together with the Gárdos channel, PIEZO1 forms in RBCs a tandem that participates in the rapid adjustment of the cell volume. The pharmacology allowing functional studies of the roles of PIEZO1 has only recently been developed, with Yoda1 as a widely used PIEZO1 agonist. In 2018, Yoda1 analogues were developed, as a step towards an improved understanding of PIEZO1 roles and functions. Among these, Dooku1 was the most promising antagonist of Yoda1-induced effects, without having any ability to activate PIEZO1 channels. Since then, Dooku1 has been used in various cell types to antagonize Yoda1 effects. In the present study using RBCs, Dooku1 shows an apparent IC_50_ on Yoda1 effects of 90.7 µM, one order of magnitude above the previously reported data on other cell types. Unexpectedly, it was able, *by itself*, to produce entry of calcium sufficient to trigger Gárdos channel activation. Moreover, Dooku1 evoked a rise in intracellular sodium concentrations, suggesting that it targets a non-selective cation channel. Dooku1 effects were abolished upon using GsMTx4, a known mechanosensitive channel blocker, indicating that Dooku1 likely targets PIEZO1. Our observations lead to the conclusion that Dooku1 behaves as a PIEZO1 agonist in the RBC membrane, similarly to Yoda1 but with a lower potency. Taken together, these results show that the pharmacology of PIEZO1 in RBCs must be interpreted with care especially due to the unique characteristics of RBC membrane and associated cytoskeleton.

## 1 Introduction

PIEZO1 is a mechanosensitive ion channel, present in most eukaryotic species (Coste et al., 2010), it has been shown to be the effector of transduction of mechanical force sensing in multiple cell types and tissues (Syeda, 2021). PIEZO1 allows the passage of mono- and divalent cations (Gnanasambandam et al., 2015) in response to mechanical signals, with two putative mechanisms for signal transduction: either via contact with the actin cytoskeleton (Wang et al., 2022) or via sensing of membrane curvature and tension (Cox et al., 2016) as recently demonstrated in mouse RBCs (Vaisey et al., 2022).

The presence of the PIEZO1 channel in human RBC membranes was first established from the link between gain-of-function mutations of PIEZO1 and a RBC pathology, termed Dehydrated Hereditary Stomatocytosis (DHS) or Xerocytosis (Zarychanski et al., 2012; Andolfo et al., 2013; Bae et al., 2013). RBCs possess a repertoire of membrane ion transporters, which maintain cell homeostasis and volume. Cation transporters represent an efficient tool for cell volume regulation. Among them, cation channels are the most efficient, given their rapid kinetics and permeability rates. Most notably, the tandem constituted by the Gárdos channel (KCNN4, KCa3.1), a Ca^2+^-activated K^+^ channel, and PIEZO1 is often cited as a key regulator of RBC volume (Lew and Tiffert, 2017; Caulier et al., 2018; Jankovsky et al., 2021); PIEZO1 appearing as a sensor and the Gárdos channel as an effector of the volume decrease accompanying deformability. Given the very small number of copies of PIEZO1 (30-80) in the RBC membrane (Gautier et al., 2018; Vaisey et al., 2022), a convenient way to observe PIEZO1 effect is through the activity of the Gárdos channel.

The unravelling of the molecular identity of PIEZO1 has allowed the development of PIEZO1-specific pharmacology; this has allowed the characterization of its role in various situations. Like most mechanosensitive channels, it is inhibited by the spider toxin GsMTx4 (Bowman et al., 2007; Bae et al., 2011). In 2015, a small synthetic molecule termed Yoda1 was described as an agonist for human and mouse PIEZO1, prominently affecting the sensitivity and the mean time of the open state of the channel (Syeda et al., 2015). Later it was proposed that Yoda1 acts by lowering the mechanical threshold for channel activation (Botello-Smith et al., 2019) or by voltage-activating PIEZO1 (Wijerathne et al., 2022). Since then, the molecule has proven to be a useful tool in studies targeting PIEZO1. In 2018, a series of Yoda1 analogues were designed and tested on the PIEZO1 channel in an attempt to expand its pharmacology (Evans et al., 2018). By modifying the pyrazine ring of Yoda1, or substituting its thiadiazole group with oxadiazole, several analogues that can reversibly antagonize Yoda1, were obtained. Among them, a compound judiciously named Dooku1 gave the most promising initial results. Based on tests either on HEK293 cells expressing human PIEZO1 or on HUVECs, Dooku1 showed no effect by itself on PIEZO1. Nevertheless, it was able to inhibit a 2 µM Yoda1-induced Ca^2+^ entry with an IC_50_ of 1.3–1.5 µM. Thus, a competitive effect on the same or similar binding site by Yoda1 was proposed (Evans et al., 2018). Since then, Yoda1 and Dooku1 have been used in combination in several cell types to decipher PIEZO1 activity and functions (Deivasikamani et al., 2019; Matsunaga et al., 2021; Barnett et al., 2022; Szabó et al., 2022), including RBCs (Wadud et al., 2020).

In the present work, we have investigated the consequences of Dooku1 addition on Yoda1-induced effects on human RBCs. To our surprise, we observed that Dooku1 itself has effects on RBC membrane potential and triggers a rise in intracellular Ca^2+^; this in turn leads to the activation of the Gárdos channel, similarly to Yoda1 but with a lower potency. We also unravel a moderate inhibitory capacity for Dooku1 on Yoda1 effects in RBCs. Finally, we show that Dooku1 is able to activate PIEZO1 within the RBC membrane.

## 2 Materials and methods

### 2.1 Reagents and drugs

All salts were acquired from Sigma and were of analytical grade or better. Dooku1: 2-[(2,6-Dichlorobenzyl)thio)-5-(1H-pyrrol-2-yl)-1,3,4-oxadiazole (Sigma-Aldrich, France). Yoda1: 2- [5-[[(2,6-Dichlorophenyl)methyl]thio]-1,3,4-thiadiazol-2-yl]pyrazine (Tocris, France). Charybdotoxin (Alomone labs, Israel). CCCP: carbonyl cyanide 3-chlorophenylhydrazone (Sigma-Aldrich, France). GsMTx4 (Alomone labs, Israel). Fluo-4, AM (Invitrogen, France). All drugs are used at 1000X stock solution in DMSO, except Charybdotoxin and GsMTx4 in water.

### 2.2 Solutions

Solution A: Buffered Ringer for water and ion content measurements: 150 mM NaCl, 2 mM KCl, 2 mM CaCl_2_, 1 mM MgCl_2_, 10 mM HEPES, 5 mM Glucose with pH 7.4 adjusted with NaOH.

Solution B: Unbuffered Calcium Ringer for membrane potential measurements using MBE method: 154 mM NaCl, 2 mM KCl, 2 mM CaCl_2_.

Solution C: Live flow cytometry and imaging solution: 137 mM NaCl, 3.5 mM KCl, 2 mM CaCl_2_, 1 mM MgCl_2_, 10 mM HEPES, 10 mM Glucose, 0.05% BSA with pH 7.4 adjusted with NaOH.

### 2.3 Ethical statement

Blood from healthy volunteers was withdrawn upon written informed consent (EFS, Etablissement Français du Sang), in accordance with the guidelines of the Helsinki declaration of 1975, as revised in 2008. This work has been approved by the institutional (CNRS) Ethical committee and by the French Ministry of Research (declaration DC-2019-3842).

### 2.4 Red blood cells

#### 2.4.1 For membrane potential measurements

Blood from healthy donors was withdrawn into heparinized vacuum tubes, washed thrice with unbuffered saline by centrifugation for 5 min at 2,500 rcf, the buffy coat and plasma removed, then packed with a final step of 1-min centrifugation at 12,000 rcf, and the packed cells stored at 4°C until used.

#### 2.4.2 For microscopy acquisition and flow cytometry

25 µL of blood from healthy donors were washed thrice with *Solution C* for 5 min at 2,500 rcf. RBCs were loaded with Fluo-4, AM at a concentration of 5 µM for 1 h at 37°C with 400 rpm shaking. Cells were rinsed thrice with the *Solution C* for 5 min at 2,500 rcf.

#### 2.4.3 For intracellular sodium, potassium, and water content

Fresh blood collected into heparinized tubes from healthy donors were washed thrice at 2,500 rcf for 5 min using the *Solution A*. A final solution at 20% haematocrit was prepared. Cells were placed in a warmed stirring incubator at 37°C with 400 rpm shaking. Drugs were added at their designed concentration. 0.5 mL aliquots of the cell suspension were taken at each timepoint, distributed in Beckman polyethylene micro test tubes (Dutscher, France) and centrifuged at 19,600 rcf for 7 min at 10°C. After centrifugation, the packed cell mass was separated from the supernatant by slicing the tube with a razor blade below the top of the red cell column prior weighting (see intracellular ionic and water section measurements).

### 2.5 Membrane potential measurement

The CCCP method or MBE method (method of Macey, Bennekou, and Egee) as it was recently named by Jansen et al., 2021(Macey et al., 1978; Bennekou and Christophersen, 1986; Jansen et al., 2021; Pérès et al., 2021) was used to monitor membrane potential evolution. Briefly, when RBCs are suspended in a nominally buffer-free solution in the presence of the CCCP protonophore (20 µM), changes in extracellular pH reflect membrane potential changes since protons are kept at equilibrium across the membrane. The membrane potential (V_M_) can, thus, be estimated from the equation:

$$V_{M}=61.51.\;\left({pH}_{i}-{pH}_{o}\right),in\;mV$$

Due to the high red cell buffer capacity, the intracellular pH (pH_i_) remains constant (at about 7.2) throughout an experiment and can be estimated as the pH of the solution after lysis with Triton™ X-100 at the end of the experiment. Regarding the experimental procedure, 2,900 µL of the experimental solution containing 20 µM of CCCP was heated at 37°C under constant magnetic stirring. For each experiment, 100 µL of packed RBCs (99% hct) were added, to reach a final cytocrit of 3.3%. All inorganic compounds were added at stock solution 1000X in DMSO, unless stated otherwise. The final DMSO concentration never exceeded 0.3%, a concentration that has no effect on either fluxes or membrane potential. Extracellular pH was measured using a G200 pH electrode (Radiometer, Copenhagen, Denmark) coupled to a red Rod 200 reference electrode (Radiometer) and a PHM210 pHmeter (Radiometer). Sampling and acquisition were done with an electrode amplifier (EA-BTA, Vernier, United States) at a rate of 1 Hz connected to an AD LABQUEST Mini interface (Vernier, United States) with a resolution of 0.01 pH unit. The data were visualized and analyzed with the Logger Lite software (Vernier, France). At the end of each experiment, Triton X-100 detergent (1% in 3M NaCl) was added, causing total cell lysis and a resulting solution that attains the intracellular pH.

### 2.6 Flow cytometry

At t = 0, 2.5 µL of Fluo-4 loaded RBCs were added to 500 µL of *solution C* containing drugs at desired concentration and immediately observed on the cytometer. Flow cytometry measurements were performed on a BD FACSCanto™ II (BD Bioscences, Erembodegem, Belgium, RECYF platform, Station Biologique, Roscoff, France). Dooku1 dose-response curve (1 μM, 10 μM, 100 µM), Yoda1 (625 nM), and Yoda1+ Dooku1 (10 µM) testing was realized on 3 independent healthy donors. For each sample, data were recorded during 5 min after starting drugs incubation. The results were assessed and analyzed using Kaluza (Beckman Coulter, Life Sciences, France).

### 2.7 Confocal microscopy

2.5 µL of Fluo-4- loaded RBCs were diluted in 300 µL of *solution C* and deposited on Polylysine-coated coverslips. At *t* = 0, 300 µL of 2X drug containing solution was added, to reach desired final concentration, and imaged on a SP8 confocal microscope (Leica-Germany) linked to a LAS-X software (Merimage platform, Station Biologique, Roscoff, France). Fluorescence analysis was done using Fiji software, *n* = 40 to 170 cells quantified for each time point (Schindelin et al., 2012).

### 2.8 Intracellular water, Na^+^, and K^+^ measurements

#### 2.8.1 Water content

After weighting, the packed cells were dried to constant weight for at least 48 h at 90°C and re-weighted. RBC volume depends on the intracellular water content, which is estimated to be about 90 fL for a healthy discocyte. Shape change can be misleading in the estimation of cellular water content due to the great plasticity of the red cell membrane. These measurements are independent of cell shape.

#### 2.8.2 Na^+^ and K^+^ content

The packed cells within the sliced tubes were lysed in 1 mL MilliQ water. Proteins were denatured to ease separation by addition of 232 µM of perchloric acid. The tubes were spun at 12,000 rcf for 7.5 min at 4°C and the supernatant was passed onto sample tubes and diluted 10 times. The ionic content was measured using a flame photometer (PFP7 Jenway, France). The amounts of Na^+^ or K^+^ measured are reported as mmol/L of cell water.

### 2.9 Statistics and data analysis

GraphPad Prism version 9 (GraphPad Software, San Diego, California United States) was used for the statistical analysis and presentation of the data. One-way ANOVA with multiple comparisons was used to test for statistical significance. Data are represented as mean ± standard deviation (SD). *p* ˂ 0.05 was deemed as significant.

## 3 Results

### 3.1 Dooku1 can trigger a Gárdos effect

**FIGURE 1:**
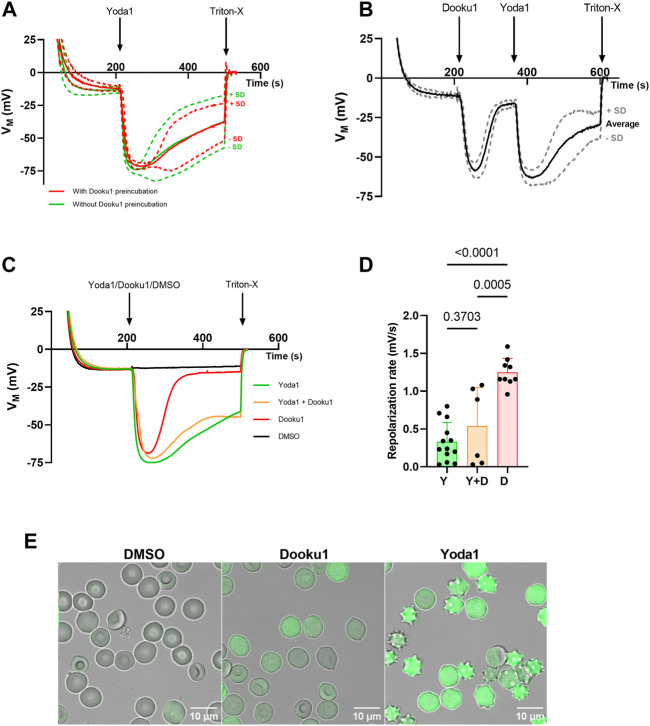
Dooku1 alone can trigger a Gárdos effect. **(A)** Evolution of RBC membrane potential after injection of Yoda1 (625 nM, green, *n* = 13) without any pre-incubation, and Yoda1 (625 nM, red, *n* = 5) after a pre-incubation for 15 min at 37°C with Dooku1 (10 µM). Mean traces are plotted with their SD envelopes **(B)** Evolution of membrane potential after challenging the cells successively with Dooku1 (10 µM) and then with Yoda1 (625 nM). Traces represent average ± SD, *n* = 6. The mean trace was plot with its SD envelope **(C)** Changes in RBC membrane potential after injection of Yoda1 (625 nM, green, n = 13), Dooku1 (10 μM, red, *n* = 9), and simultaneously Yoda1 (625 nM) and Dooku1(10 μM, orange, *n* = 6). The black line represents control done with vehicle alone (DMSO, *n* = 4). Traces represent average (SD envelope is not drawn for sake of clarity). **(D)** Repolarization rate in different conditions measured right after maximum hyperpolarization. The repolarization values were obtained by calculating the derivative values between two-time points during which the repolarization takes place. Histograms represent means ± SD for each condition. **(E)** Confocal microscopy images of Fluo-4-loaded RBC 1 min after perfusion of DMSO 0.1% (left), Dooku1 (10 μM; middle) or Yoda1 (625 nM; right).

Yoda1 is the most efficient chemical agonist of the PIEZO1 channel identified thus far, leading to calcium entry and eventually Gárdos channel opening once its threshold of activation is reached. Gárdos channel current strength is strictly dependent on intracellular calcium concentration (channel opening probability). This results in noteworthy K^+^ efflux and hyperpolarization (Figure 1A; Supplementary Figure S1A). Dooku1 has been described recently as an antagonist of this effect (Evans et al., 2018; Wadud et al., 2020). In our observations, contrary to what was described by others, preincubation of RBCs with Dooku1 (10 µM) did not affect neither Yoda1-triggered hyperpolarization (Figure 1A) nor repolarization rate (Figures 1B–D). Even more surprisingly, Dooku1 alone induces hyperpolarization, to the same extent as observed with Yoda1 (Figures 1B, C; Supplementary Figure S1B). This result indicates that Dooku1 can provoke a sufficient entry of Ca^2+^ within RBCs to activate the Gárdos channel. In the case of Dooku1, however, the repolarization rate is faster than with Yoda1 (Figures 1C, D), suggesting that the amount of calcium entry triggered by Dooku1 is lower than with Yoda1. This was verified by microscopy on Fluo-4 loaded RBCs, where Ca^2+^ entry after Dooku1 perfusion can clearly be seen; the magnitude is demonstrably lower than with Yoda1 (Figure 1E). To ensure that the observed hyperpolarizations were indeed due to Gárdos channel activation, Gárdos was inhibited with 100 nM of Charybdotoxin (ChTX) prior to addition of Dooku1 and Yoda1 addition. In the presence of ChTX, all hyperpolarization was abolished (Supplementary Figure S1C).

### 3.2 Dooku1, an activator as well as an antagonist of Yoda1

**FIGURE 2:**
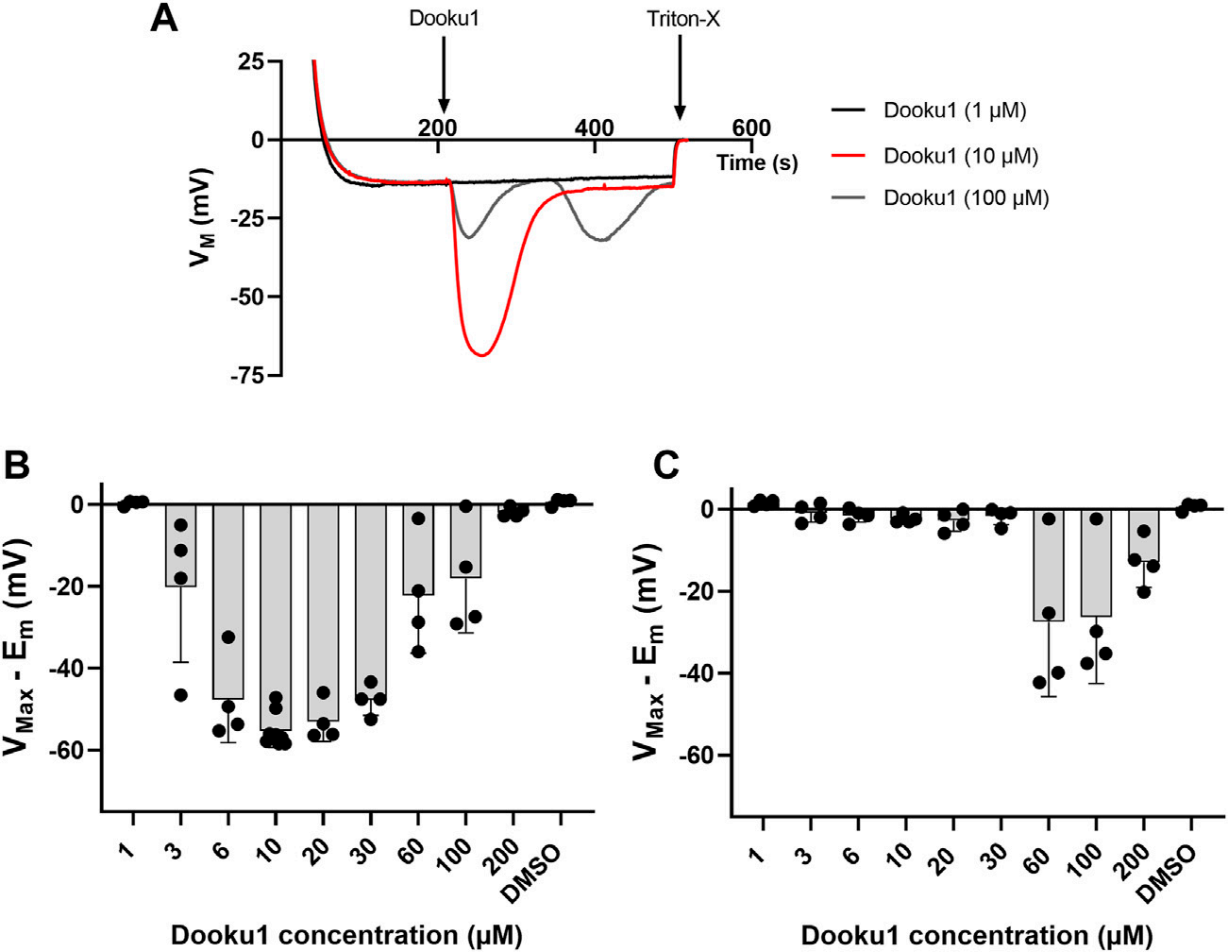
Dooku1 dose-response curve. **(A)** Variations in membrane potential after addition of Dooku1 at different concentrations: 1 µM (black trace), 10 µM (red trace), and 100 µM (grey trace). Traces represent means of *n* = 4 independent replicates for each condition. SD envelopes were not added for clarity reasons **(B,C)** Maximal hyperpolarizations induced by increasing concentrations of Dooku1. Histograms represent the maximal hyperpolarizations (V_Max_) that were reached, from which were deduced the nominal resting membrane potential (E_m_). In **(B)** values of the first hyperpolarization occurring immediately after Dooku1 injection; and in **(C)** values of the second hyperpolarization, when occurred, which was delayed in time. Histograms represent means ± SD of *n* = 4 experiments.

Next, a dose-response study of the effects of Dooku1 on erythrocyte membrane potential was performed. Between 1 and 10 μM, there was a direct dose-dependent effect (Figures 2A, B), with an increased maximal hyperpolarization observed immediately after Dooku1 injection. With a Dooku1 level of 3 μM, Dooku1-induced hyperpolarization was significant (*p* = 0.014 for 3 μM, *p* < 0.0001 for 6 µM–30 μM, *p* = 0.0057 for 60 μM, *p* = 0.0326 for 100 μM, *n* ≥ 4) compared to the vehicle control (DMSO). However, at 200 µM of Dooku1, the value of hyperpolarization attained was not significant; it was close to that obtained with 1 µM (*p* = 0.999). Since maximum hyperpolarization is related to Gárdos channel activity which is only dependent on [Ca^2+^]i, this suggests that Dooku1 allows a dose-dependent calcium entry.

Furthermore, at concentrations greater than 30 μM, the response to Dooku1 became more complex: i) a progressive decrease in the hyperpolarization amplitude appeared and ii) a delayed second hyperpolarization which occurred 55 ± 17 s (*n* = 11) after the end of the first hyperpolarization, irrespective of the concentration used (Figures 2A, C). However, the magnitude of this second hyperpolarization decreased at Dooku1 concentrations between 60 and 200 µM (Figure 2C). We hypothesize that the poorly observed solubility of the Dooku1 molecule at high concentrations, as reported by Evans et al., 2018, explains this unexpected but reproducible phenomenon (See Section 4).

**FIGURE 3:**
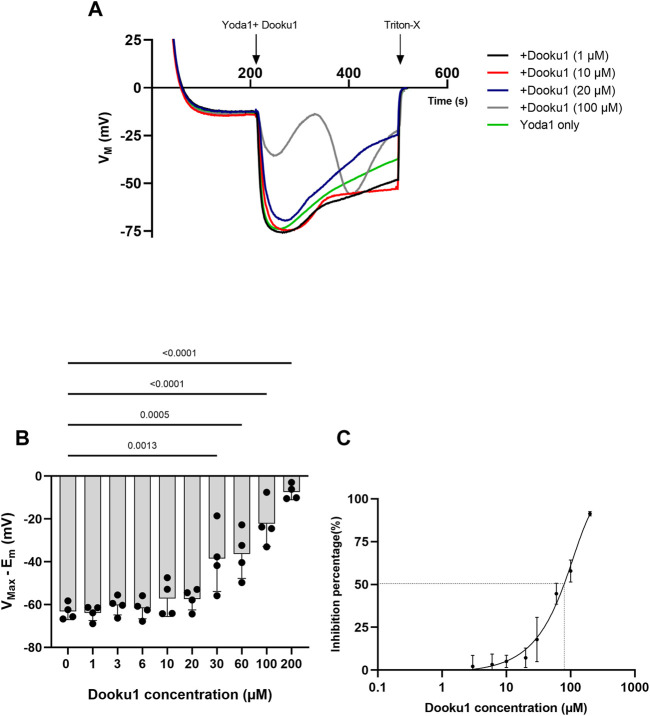
Dooku1 inhibitory effects on Yoda1-induced hyperpolarizations **(A)**Variations in membrane potentials in response to Dooku1 at different concentrations 1 µM (black trace), 10 µM (red trace), 20 µM (blue trace), and 100 µM (grey trace), added simultaneously with Yoda1 (625 nM). Yoda1 alone (green trace). Traces represent means of 4 independent experiments for each condition. SD envelopes were not added for clarity reasons **(B)** First maximal hyperpolarization observed with increasing Dooku1 concentrations, added simultaneously with Yoda1 (625 nM). Histograms represent means ± SD (*n* = 4), **(C)** Dose-response curve of Yoda1-induced hyperpolarizations inhibition by Dooku1 (*n* = 3) according to the first hyperpolarization only.

We also tested the dose-response of Dooku1 inhibition on Yoda1 effects on RBC membrane potential by adding the two drugs simultaneously. Below 20µM, Dooku1 was unable to block Yoda1-induced hyperpolarization (Figures 3A, B). At 20 μM, a slight, but not statistically significant inhibition was observed. It became significant at 30 µM (17.9% ± 12.9%, *n* = 4, *p* = 0.0013), reaching 91.3% ± 1.26% (*n* = 4), *p* < 0.001, and at 200 µM with an IC_50_ of 90.7 ± 10.7 µM (*n* = 3, Figures 3B, C).

### 3.3 Dooku1 induces an increase in intracellular calcium

**FIGURE 4:**
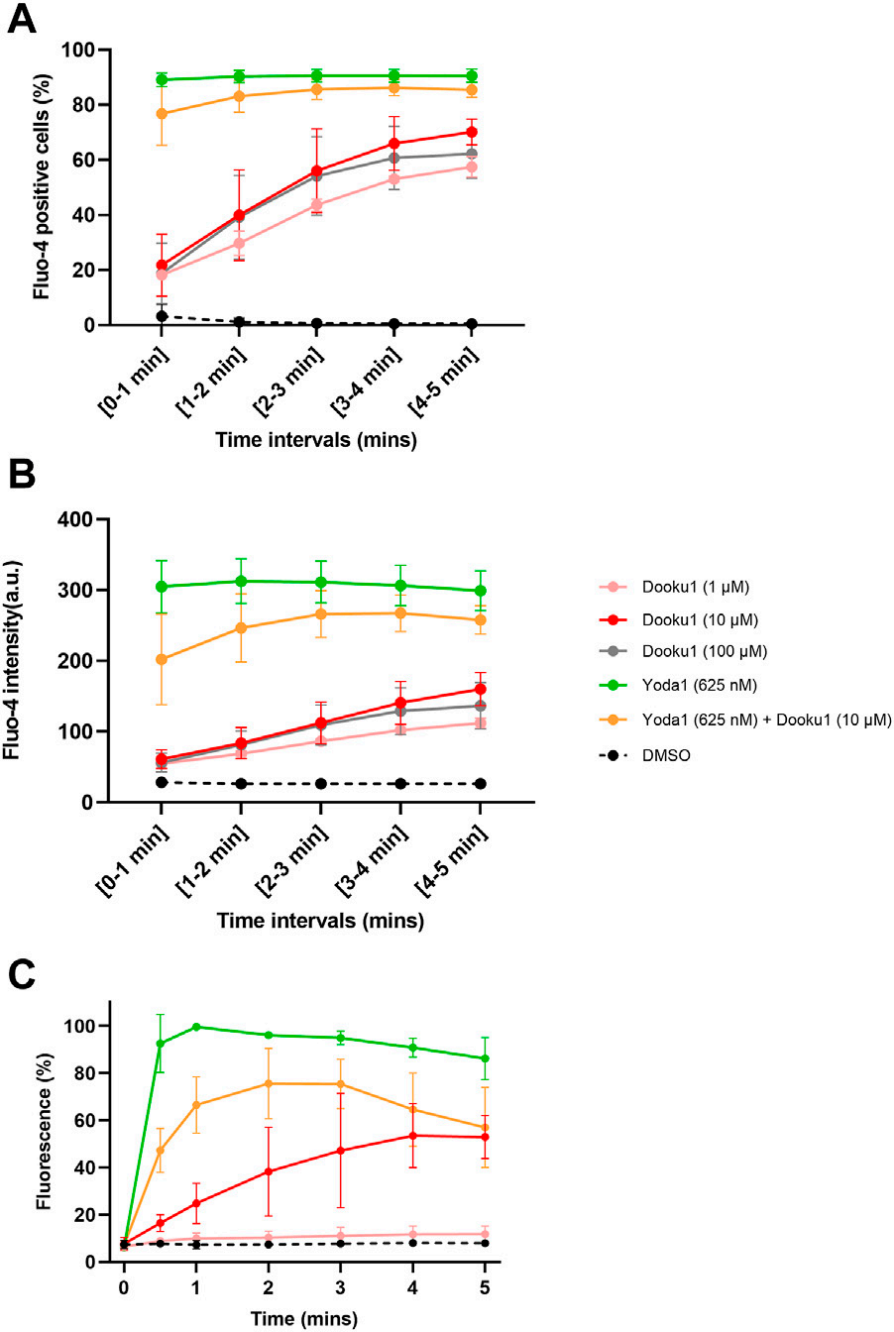
Dooku1 can generate an increase in intracellular calcium. **(A,B)** Flow cytometry follow-up of Fluo-4-loaded RBCs, measuring the percentage of Fluo-4 positive cells **(A)** and the mean fluorescence intensity (MFI) **(B)** during 5 min of Dooku1, Yoda1 or both molecules perfusion. Traces are means ± SD of *n* = 3 independent experiments. **(C)** Mean fluorescence of Fluo-4-loaded RBCs in confocal imaging, after perfusion with Dooku1, Yoda1 or both. Mean fluorescence of RBCs was calculated and are expressed as percentages of Yoda1-induced maximum fluorescence. Data are averages ±SD of *n* = 3 independent experiments.

Given that hyperpolarization can be fully inhibited by ChTX (100 nM, Supplementary Figure S1C), it can be assumed that this phenomenon is driven by Gárdos channel activation. Thus, this result suggests that prior to Gárdos activation, [Ca^2+^]_i_ reaches the threshold for increasing the open probability of the latter channel. To assess that Dooku1 is able to induce a calcium entry by itself, both imaging and flow cytometry were performed. Cells were loaded with 5 µM of Fluo-4 to detect cells with increased intracellular calcium concentration. Cells were perfused with Dooku1 and/or Yoda1, and fluorescence was measured during a 5-min interval. Yoda1 (625 nM) evoked an instantaneous increase in intracellular calcium in 89.1% of the cells, showing that Yoda1 has induced an immediate intracellular entry of calcium. On the contrary, Dooku1 at 10 µM induced a progressive calcium entry, with only 21% of the cells showing increased intracellular calcium after 1 min, to reach 70.1% after 5 min. Interestingly, at this concentration, simultaneous perfusion of both Dooku1 and Yoda1 delayed the entry of calcium (only 76.79% of Fluo-4 positive cells after 1min and 85.5% of Fluo-4 positive cells at 5 min) (Figure 4A).

In addition, the mean fluorescence intensity recorded upon Dooku1 stimulation either by flow cytometry or microscopy imaging indicates that the mean fluorescence reached only 20% of the one obtained with Yoda1 after 1 min (Figures 4B, C). As for the number of Fluo-4 positive cells, progressively mean fluorescence intensity extends to reach around 53% (53.5% for flow cytometry and 52.9% with confocal microscopy) of the one obtained with Yoda1 after 5 min. When Dooku1 was added simultaneously with Yoda1, it was able to delay the load of Ca^2+^ (Figures 4B, C). Essentially, Dooku1 can induce a significant calcium entry by itself even at 1 μM, as seen with flow cytometry (Figures 4A, B) and confocal microscopy (Supplementary Figure S2).

Taken together, these results show that Dooku1 can trigger a calcium entry, by itself, albeit more slowly and with a lower extent than Yoda1 but sufficient to induce a Gárdos channel activation.

### 3.4 Dooku1 activates a non-selective cation channel

It has been widely reported that the activation of PIEZO1 by Yoda1 induces a cell dehydration accompanied by a reversal of sodium and potassium gradients due to its nature of being a non-selective cation channel (Monedero Alonso et al., 2021; Pérès et al., 2021).

**FIGURE 5:**
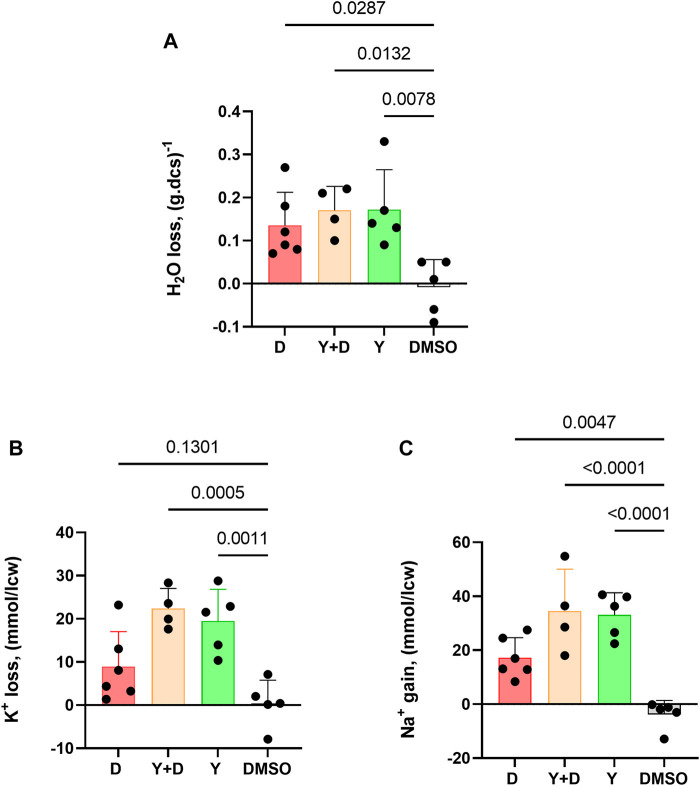
Dooku1 can alter the intracellular water, K^+^, and Na^+^ contents of the RBC. Measurements of **(A)** Water content loss **(B)** K^+^ loss and **(C)** Na^+^ gain after 30 min of incubation with Dooku1 (10 µM), Yoda1 (625 nM) or both. Histograms represent means ± SD of *n* = 6 for Dooku1 (D, in red), *n* = 4 for Yoda1+Dooku1 (Y + D, in orange), *n* = 5 for Yoda1 only (Y, in green), and *n* = 5 DMSO (black).

The impact of Dooku1 and Yoda1 molecules on the hydration status and ion contents of the cells has been evaluated. Dooku1, Yoda1, or both molecules added simultaneously, all induced water loss reiterating the results that these molecules trigger a Gárdos effect that leads to dehydration (Figure 5A).

Gárdos channel activation is accompanied by K^+^ loss. Compellingly, K^+^ effluxes generated using Dooku1 were smaller than in the presence of Yoda1. This result is in accordance with previous results presented here, in which we demonstrated that Dooku1 leads to [Ca^2+^]_i_ rise, but with an intensity lower than that observed with Yoda1. As a result, Gárdos channel is logically less activated with Dooku1, resulting in lower K^+^ loss (Figure 5B).

**FIGURE 6:**
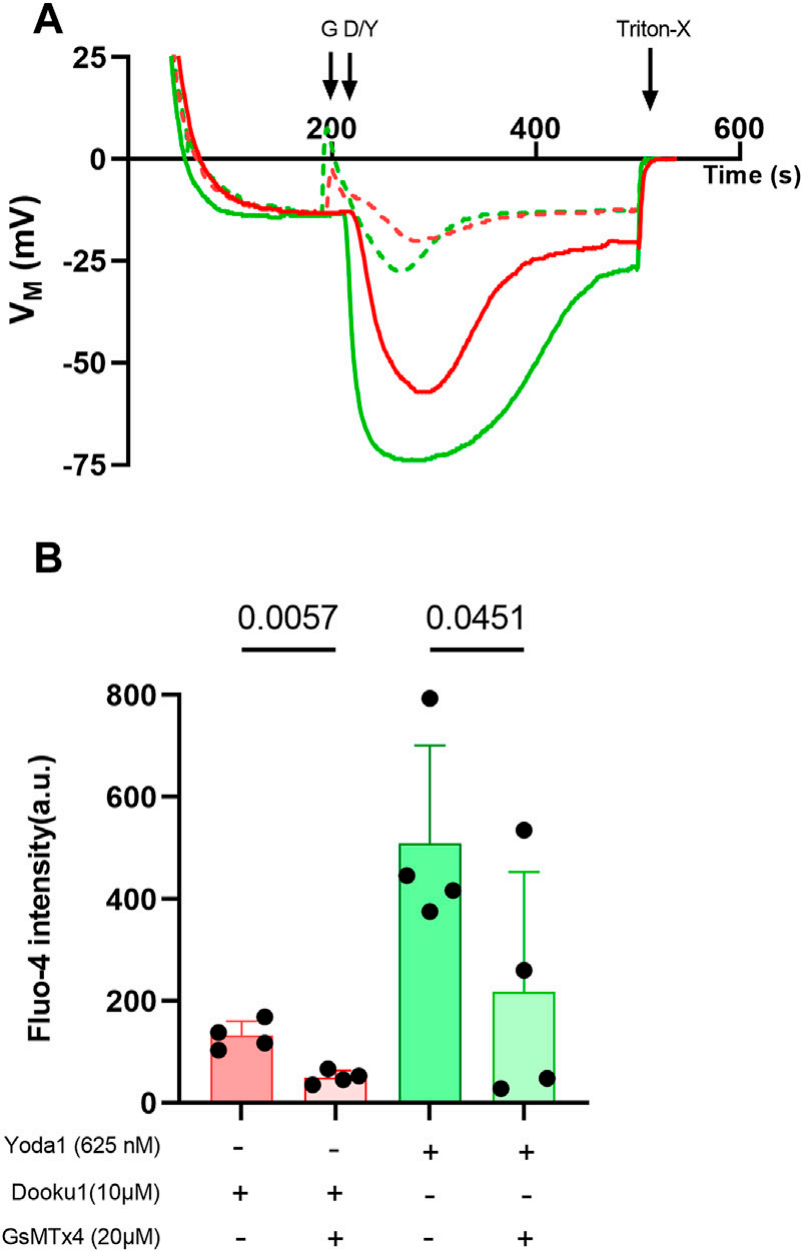
GsMTx4 can inhibit Dooku1 effects. **(A)** A representative trace showing the partial inhibition of membrane potential variations after using GsMTx4 (G). Cells were preincubated with GsMTx4 (20 µM) for 20 min at 37°C, then GsMTx4 (20 µM) was added to the medium at 190 s. Cells were challenged with Dooku1 (10 μM, in red; plain line without GsMTx4, dotted line with GsMTx4) or Yoda1 (625 nM, in green; plain line without GsMTx4, dotted line with GsMTx4) at 210 s. **(B)** Comparison of Fluo-4 intensity observed by flow cytometry. Histograms represent means ± SD (*n* = 4). Statistical differences were assessed using paired one-way ANOVA.

Finally, Dooku1, Yoda1, and both molecules added together, all induced significant [Na^+^]_i_ increase (Figure 5C). This result suggests that both molecules activate a non-selective cation channel, and both have the same target, presumably PIEZO1. The effects of Dooku1, as for Yoda1, were inhibited by GsMTx4, supporting the latter hypothesis (Figure 6).

## 4 Discussion and conclusion

Deciphering non-selective cation permeability in erythrocytes, and PIEZO1 behavior in particular, is essential to understand the hydration state of the cells in physiological and pathophysiological conditions (Gallagher, 2017; Caulier et al., 2018; Evans et al., 2020; Yamaguchi et al., 2021). Pharmacological tools are a pre-requisite for these studies. Yet, PIEZO1 pharmacology is still at its infancy as stated by Evans *et al* (Evans et al., 2018). To address this deficit, Evans and coworkers designed Dooku1, a Yoda1 analogue that lacks any agonist effect on PIEZO1.

The present study shows unambiguously the capacity of Dooku1, by itself, to activate a Ca^2+^ permeability in human RBC membrane that is able to turn on the Gárdos channel. MBE measurement on a RBC population, flow cytometry or confocal imaging of individual RBCs all gave proof that in the human RBC PIEZO1 is sensitive to Dooku1.

The human RBC has unique characteristics, that allow a tremendous capacity to undergo the required deformations to flow and bend into the narrowest of the capillaries. The first relates to the cell’s surface area to volume ratio and the second to the membrane deformability. This membrane deformability relies on a submembranous cytoskeleton composed of a network of spectrin filaments bounds to nodes connected to transmembrane proteins (Fowler, 2013), and a unique lipid composition with one of the highest cholesterol/phospholipid ratio (Subczynski et al., 2017). Moreover, mechanosensitivity of the PIEZO1 channel is strictly dependent on lateral tension and curvature that act as the mechanosensory stimulus to trigger channel openings and deactivation. These two parameters are themselves determined by lipid composition (Božič and Svetina, 2022; Vaisey et al., 2022). PIEZO1 can also be activated with voltage changes (Kaestner and Egee, 2018; Moroni et al., 2018). Altogether, these characteristics make a very special environment for PIEZO1, such that its gating in RBCs is probably specific. Indeed, a report on mouse RBCs has already shown that this specific environment impairs the rapid inactivation mechanism of PIEZO1 that was observed in other cell types, shedding light on a slower deactivation mechanism in RBCs that could be modified upon PIEZO1 mutations (Evans et al., 2020). Similarly, it has been demonstrated that gain-of-function mutations of PIEZO1, reported as deleterious in RBCs, lose their phenotypes once heterologously expressed in HEK293 cells (Yamaguchi et al., 2021).

Even the pharmacology of PIEZO1 can be affected by surrounding composition, since the effects of Yoda1 in RBCs activates the channel without mechanical stimulation, whereas in other cell types, Yoda1 acts by increasing the sensitivity to membrane stretch (Lacroix et al., 2018; Botello-Smith et al., 2019). The elegant study of Wijerathne et al., 2022, has biophysically characterized the effects of Yoda1 on the activation of PIEZO1 in HEK293 cells, revealing that Yoda1 activates the channel by energetically stabilizing and destabilizing its conducting and non-conducting parameters (Wijerathne et al., 2022).

In the current study, we have shown that a drug, Dooku1, that has no effects on PIEZO1 in other cell types is able to activate the channel in human RBCs at rest (1–3 µM). Further, Dooku1 shows only mild antagonizing effects in RBC (IC_50_ 90.7 µM) compared to those described in other cell types such as HEK293 (IC_50_ 1.3 µM) or HUVEC (IC_50_ 1.5 µM) (Evans et al., 2018). Consistent with these results, Dooku1 would be expected to have a competitive antagonism effect on the Yoda-1 binding site rather than directly blocking the channel (Evans et al., 2018). Our present results sustain this hypothesis. The study of Wijerathne *et al*, despite being having been performed in HEK cells, corroborates this hypothesis since they clarified that Dooku1 has an effect on the kinetics of the channel, an effect too small to affect the open probability and the macroscopic behavior of the channel, therefore Dooku1, at 30 μM, acts as a silent binder on PIEZO1 (Wijerathne et al., 2022).

The MBE method is rapid and accurate for studying ion channel regulation and pharmacology (Monedero Alonso et al., 2021). Membrane potential variations towards electronegative values indicate a K^+^ efflux, which translates into Gárdos channel activation. Via the MBE method, hyperpolarizations speeds and magnitudes recorded with Dooku1 or Yoda1 were comparable. This implies that in both cases, the Ca^2+^ threshold required for full Gárdos activation was reached rapidly. However, the pace of repolarization was faster upon Dooku1 stimulation, indicating by such that Ca^2+^ entry is probably lower in magnitude, facilitating Ca^2+^ removal by the calcium pump, and thus more quickly reducing the open probability of the Gárdos channel. Another mechanism behind the faster repolarization could be the special PIEZO1 potential-dependent conformational rearrangement in response to Dooku1, as was described by Wijerathne and colleagues, resulting in a higher probability of repetitive Dooku1 binding and in a faster repolarization (Wijerathne et al., 2022). This assumption is strengthened by flow cytometry and confocal microscopy measurements of calcium content.

The dose-response curves established in this present study (flow cytometry and imaging) show that Dooku1, even at 1 μM, evokes significant Ca^2+^ entry (Figure 4; Supplementary Figure S2). Nevertheless, hyperpolarizations could only be detected from 3 to 6 µM. This apparent discrepancy is due to a particularity of MBE method. Albeit fast and accurate, MBE method is an indirect measurement of membrane potential through the measurement of extracellular pH in an unbuffered extracellular medium. Thus, in that case the extent of extracellular pH change depends on the simultaneity of all cells to respond to the stimulation at the same pace. In the present case, at low Dooku1 concentrations (below 3 µM), a limitation of the method occurs. Moreover, both MBE method and flow cytometry, induce shear stress that are not fully comparable. So, it is possible that the difference lies also in this slight but not negligible difference considering the mechanosensitivity of Piezo1.

Unlike our results, Wadud *et al* showed in Sickle RBCs that Dooku1 at 10 µM neither increases Ca^2+^ content nor favors phosphatidylserine PS exposure. On the contrary, they demonstrated a significant but partial inhibition of the consequences of calcium increase triggered by Yoda1 (Wadud et al., 2020). These differences in results could originate within the well-known more pronounced rigidity and lack of deformability of Sickle RBCs (Gutierrez et al., 2021). Our preliminary data about Dooku1 on sickle RBCs showed lower hyperpolarization amplitude and Ca^2+^ increase (unpublished data).

Interestingly, in a series of experiments using the efficacious concentration of 10 µM of Dooku1, cells underwent significant Na^+^ increase, K^+^ loss, and dehydration (Figure 5). Whereas K^+^ loss can be related to Gárdos channel activity, Na^+^ entry indicates that Dooku1 activates a non-selective cation channel. This last result, associated with the strong inhibition of Ca^2+^ entry by GsMTx4, points out towards a direct effect of Dooku1 on PIEZO1.

Surprisingly, above 60 μM, Dooku1 induced two successive and reproducible hyperpolarizations. Our suggestion is that Dooku1 molecules precipitate at high concentrations. These precipitates could solubilize progressively over time. Due to its lipophilic nature, the insertion of Dooku1 in the lipid bilayer is likely progressive and as a consequence permits the diffusion of the compound in the solution as crystals.

In this study we highlighted the activator capacity of Dooku1. It is a realistic hypothesis that this agonist effect might be observed in RBCs only, given the particularities of PIEZO1 environment in RBC membrane. Still, the need to develop new pharmacological tools to study the non-selective cation channels (PIEZO1 indeed) is urgent: the inhibitors available so far - GsMTx4 toxin from *Grammastola Spatulata* venom, Ruthenium Red (Syeda et al., 2015) or gadolinium Gd^3+^ (Matsunaga et al., 2021)—are not specific for PIEZO1 (Bowman et al., 2007).

From our studies reported here, we can conclude that Dooku1, a molecule that is considered as an antagonist of Yoda1 without any agonist effects of PIEZO1, is sufficient to activate PIEZO1 in the RBC membrane, whilst simultaneously maintaining, at least partially, its antagonist capacities towards Yoda1. Knowing the potency of both drugs allows the opportunity to investigate PIEZO1-linked channelopathies with subtle tools (Dooku1 and Yoda1).

## Data Availability

The raw data supporting the conclusion of this article will be made available by the authors, without undue reservation.
