# Supplementary material for: Dual action of Dooku1 on PIEZO1 channel in human red blood cells
Source: Front Physiol. 2023 Jul 10;14:1222983. doi: 10.3389/fphys.2023.1222983 (PMC10365639; doi:10.3389/fphys.2023.1222983)
Supplement: Supplementary file 1 [file Table1.DOCX]

Supplementary Material

**Dual action of Dooku1 on PIEZO1 channel in human red blood cells**

**Aline Hatem^1,2^, Gwendal Poussereau^1^, Martin Gachenot^3^, Laurent Pérès^1,2^, Guillaume Bouyer^1,2^, Stéphane Egée^1,2^***

*** Correspondence:** Pr. Stéphane EGEE, egee@sb-roscoff.fr

# Supplementary Figures and Tables

## Supplementary Figures


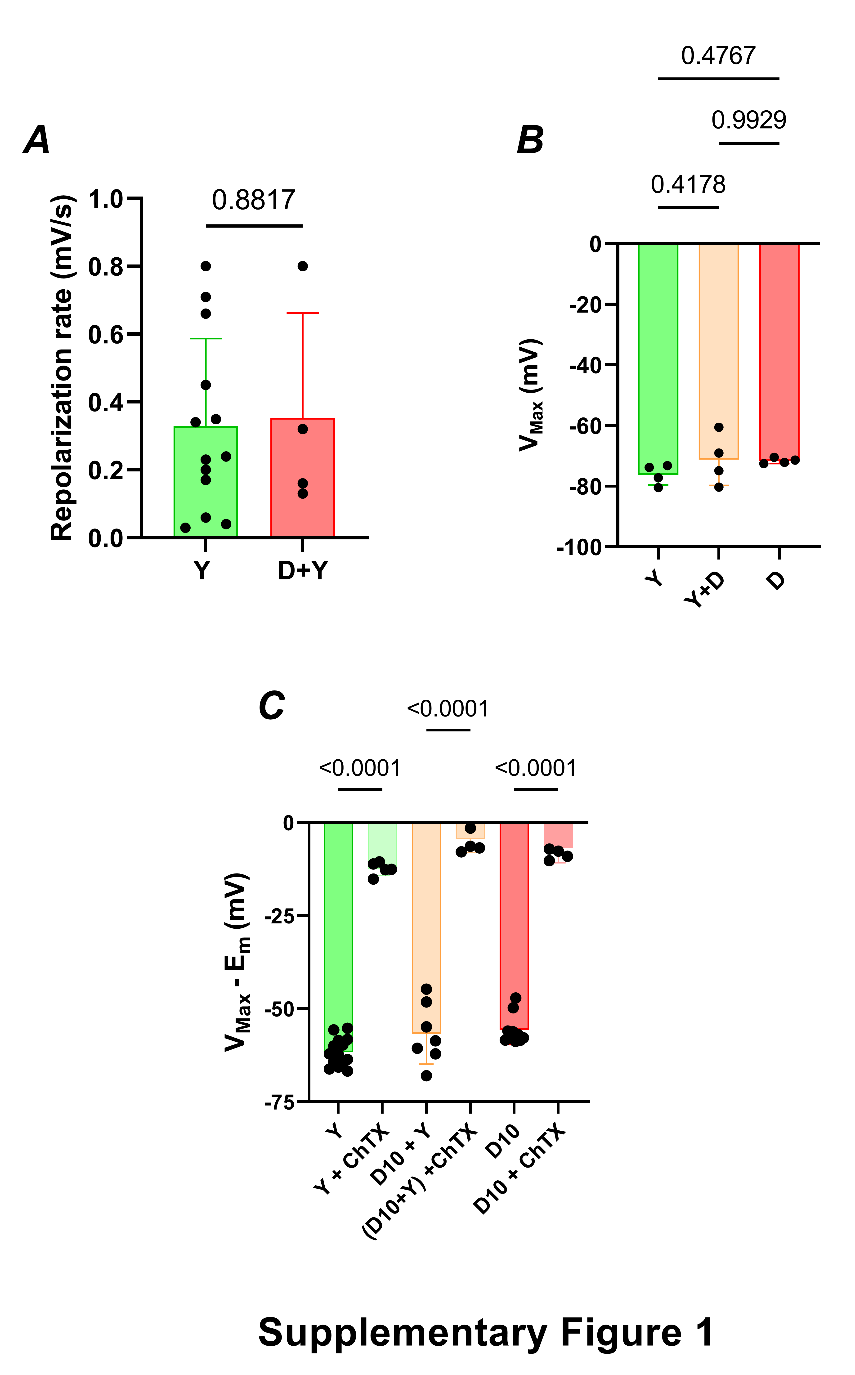


**Supplementary Figure 1.** (**A**) Repolarization rate comparison between Yoda1 only condition (green) and Yoda1 after preincubation with Dooku1 (10 µM, red) at 37°C for 15 minutes. (**B**) Maximum hyperpolarization comparison (corresponding to Fig 1C, same n) (**C**) Membrane potential variation comparison with and without inhibition with charybdotoxin (ChTX, 100 nM, n=5) Charybdotoxin was added to the cell 10s seconds before hyperpolarizing drugs addition. Each point represents the mean ±SD (same n for each condition as previously mentioned).


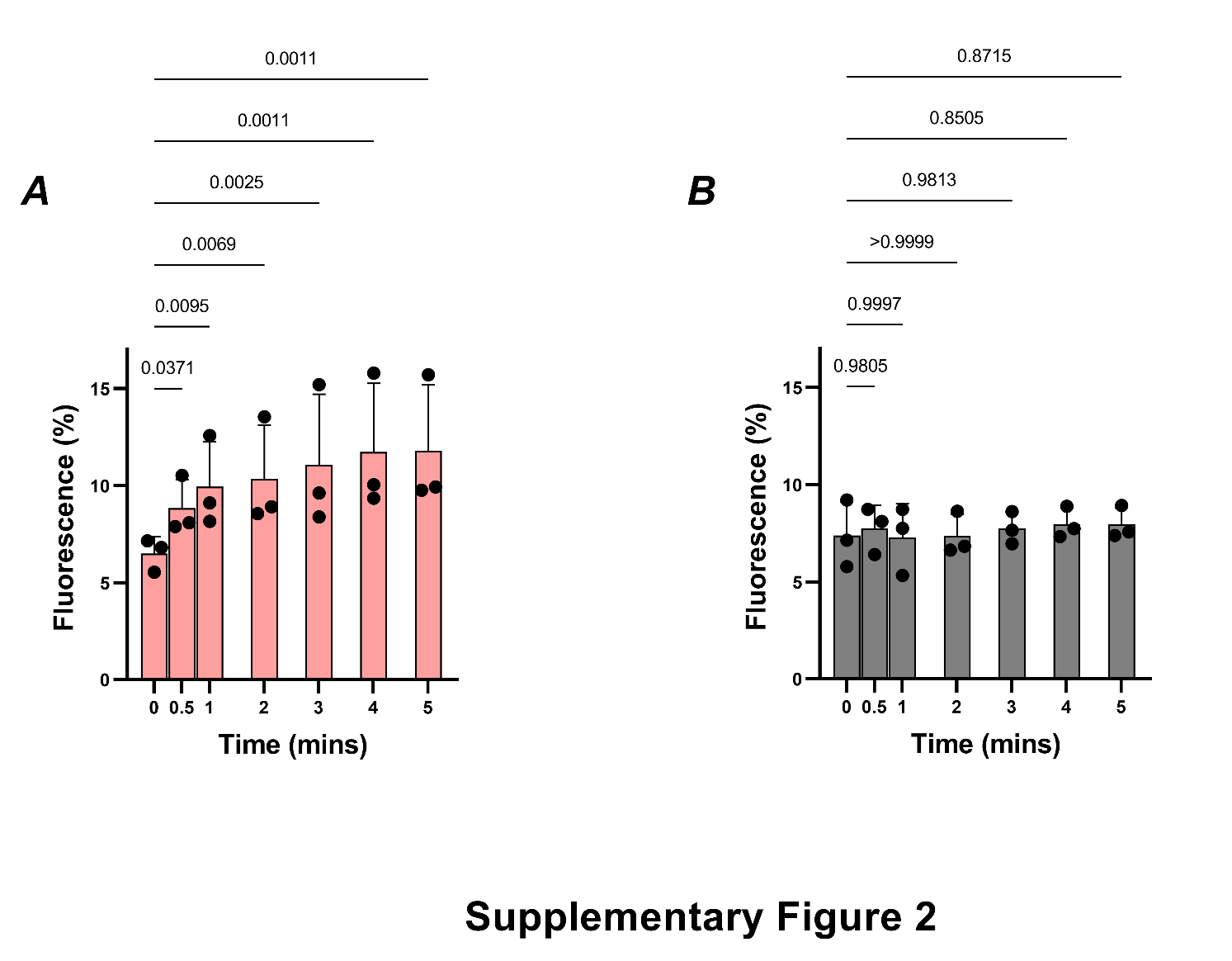


**Supplementary Figure 2.** Confocal imaging of Fluo4-loaded RBCs perfused with 1µM Dooku1 (**a**) or DMSO (**b**). Fluorescence is expressed as a percentage of Yoda1-induced maximal intensity. Histograms are means ±SD of 3 independent experiments.
